# Defining Essentiality Score of Protein-Coding Genes and Long Noncoding RNAs

**DOI:** 10.3389/fgene.2018.00380

**Published:** 2018-10-09

**Authors:** Pan Zeng, Ji Chen, Yuhong Meng, Yuan Zhou, Jichun Yang, Qinghua Cui

**Affiliations:** School of Basic Medical Sciences, MOE Key Lab of Cardiovascular Sciences, Department of Biomedical Informatics, Department of Physiology and Pathophysiology, Centre for Noncoding RNA Medicine, Peking University, Beijing, China

**Keywords:** essentiality, protein-coding genes, lncRNAs, prediction, machine learning

## Abstract

Measuring the essentiality of genes is critically important in biology and medicine. Here we proposed a computational method, GIC (Gene Importance Calculator), which can efficiently predict the essentiality of both protein-coding genes and long noncoding RNAs (lncRNAs) based on only sequence information. For identifying the essentiality of protein-coding genes, GIC outperformed well-established computational scores. In an independent mouse lncRNA dataset, GIC also achieved an exciting performance (AUC = 0.918). In contrast, the traditional computational methods are not applicable to lncRNAs. Moreover, we explored several potential applications of GIC score. Firstly, we revealed a correlation between gene GIC score and research hotspots of genes. Moreover, GIC score can be used to evaluate whether a gene in mouse is representative for its homolog in human by dissecting its cross-species difference. This is critical for basic medicine because many basic medical studies are performed in animal models. Finally, we showed that GIC score can be used to identify candidate genes from a transcriptomics study. GIC is freely available at http://www.cuilab.cn/gic/.

## Introduction

Essential genes constitute a small fraction in a genome of an organism. However, these genes underpin numerous core biological processes and are indispensable for cell viability. Insufficient expression of essential genes will lead to increased vulnerability and loss-of-function mutations of essential genes often cause lethal phenotypes (Korona, 2011; Peters et al., 2016). Essentiality is often context dependent and there also exists global essentiality (Bartha et al., 2018). Moreover, gene essentiality is not binary but has relative degree of importance in its nature (Rancati et al., 2018). Hence the classification of genes as either essential or non-essential and defining gene essentiality score has a profound influence on the study of molecular basis of various biological process (Wang et al., 2015), disease genes, drug targets, and genome design (Liu et al., 2015). In recent years, efficient gene knockout or knockdown by CRISPR/Cas9 and RNAi have been widely used to systematically evaluate the essentiality of genes and lncRNAs (Evers et al., 2016; Morgens et al., 2016) in whole organisms (Peters et al., 2016) and human cells (Wang T. et al., 2014; Wang et al., 2015; Zhou et al., 2014; Blomen et al., 2015; Zhu et al., 2016). These studies provided great helps in identifying functionally important genes and thus have great potential in discovering new genes for disease therapy and diagnosis (Tzelepis et al., 2016). However, the problem is that it is hard to apply these techniques to mammals in a large-scale. Especially, these techniques are not applicable for the whole human and therefore are often used in specific human cells.

Meanwhile, computational methods have been developed as an effective complement to predict essential genes/proteins based on protein-protein interaction (PPI) network (Gatto et al., 2015; Li et al., 2015, 2017; Zhao B. et al., 2016), ORF (open reading frame) sequence (Guo et al., 2017), and molecular evolution (Wei et al., 2013). However, these methods require attributes discriminating essential genes, e.g., conservation, gene ontology (GO) annotation and interaction network topological properties, which are only available for part of protein-coding genes. A more serious problem is that these methods often fail to predict the essentiality of long noncoding RNAs (lncRNAs), a big class of RNA molecules identified recently in human genome. The reason is that information needed by these methods is usually unavailable because most human lncRNAs show low sequence conservation and un-dissected interactions (Iyer et al., 2015). More importantly, a dataset of essential lncRNAs is still not available.

To overcome the significant limitations of current computational methods, we developed GIC (Gene Importance Calculator), an algorithm that can efficiently quantify the essentiality of both protein-coding genes and lncRNAs. Compared with previous computational methods, GIC showed competitive performance in quantifying essentiality of protein-coding genes. More importantly, GIC work well on lncRNAs but traditional methods failed. Finally, we showed the value and usefulness of GIC by three case studies. GIC web server and the source code is freely available at^1^.

## Materials and Methods

### Datasets of RNA Sequences

We downloaded human (GRCh37/hg19; Nov 9, 2014) and mouse (GRCm38/mm10; Jan 8, 2015) mRNA sequences deposited in the UCSC Table Browser (Karolchik et al., 2004). Human and mouse lncRNA transcripts were downloaded from the NONCODE database (Zhao Y. et al., 2016) (version 4) and the sequences longer than 200 nt were retained.

### Datasets of Essential Genes

We retrieved human and mouse essential protein coding genes from DEG (Luo et al., 2014) (version 10). In addition, we collected seven mouse essential lncRNAs and seven non-essential lncRNAs with experimental evidence as an independent testing set. These lncRNAs were annotated according to the Mouse Genome Informatics (MGI) database (Bello et al., 2015; Bult et al., 2016)^2^ and the results from Sauvageau et al.’s assays (Sauvageau et al., 2013). Gene CRISPR/Cas9 scores in the KBM7 cell line were obtained from Wang et al.’s study (Wang et al., 2015).

### RNA Sequence Features

The first and most basic one is RNA sequence length. Next, using a 3-nt sliding window with a step size of 1 nt, we counted the number of times each of the 64 nucleotide triplets (e.g., ACT, GCC) occurred *c*_i_ and converted it to frequency *f*_i_ by the following formula.

$$f_{i}=\frac{c_{i}}{\Sigma_{i=1}^{64}c_{i}},i=1,2,...,64$$

It should be noted that we also tried two-base code (16 codes) and four-base code (256 codes) but they showed worse performance than the triplet-base code.

Besides, we used RNAfold (Hofacker et al., 1994) (version 1.8.5) to predict RNA secondary structure with default parameters and calculate the minimum free energy (MFE) of the secondary structure. Given that longer RNAs favor lower energy state, we introduced here normalized MEF (nMFE) as follows,

$$nMFE=\frac{MFE}{L}$$

where *L* is RNA sequence length. We then mapped the RNA sequence features to their corresponding genes. For genes with multiple transcripts, the mean value was used. The ID mapping files was retrieved from the Ensembl database (Yates et al., 2016) (release 83) with the R/Bioconductor package biomaRt (Durinck et al., 2009) and manually curated.

### Logistic Regression Model and GIC Score

To reduce the number of features, especially nucleotide triplet features, we ranked the nucleotide triplet features according to their individual AUC and retained only the top five nucleotide triplet features (CGA, GCG, TCG, ACG, TCA; the same for both human and mouse) without severe co-linearity problem (Pearson correlation < 0.8) with other nucleotide triplet features. Moreover, considering that negative samples greatly outnumbered positive samples in the training set, a subset of negative samples was randomly selected to keep a 1:1 positive-to-negative ratio in the training dataset. Nevertheless, all negative samples were retained in the testing datasets in order to reflect the realistic performance of GIC score. After that, logistic regression models were constructed and cross validated for human and mouse genes and mouse lncRNAs separately. The logistic regression model is that

$$\theta (p)=\beta_{0}+\beta_{1}L+\beta_{2}nMFE+\Sigma \beta_{i}f_{i},i=\text{CGA, GCG, TCG, ACG, TCA}$$

$$where\theta (p)=\log it\left(p\right)=In\frac{p}{1-p}$$

*β*s are the coefficients of corresponding model and *p* is the conditional probability that a gene is essential (*Y* = 1). Accordingly, we defined the GIC score as the probability output *p* of the corresponding logistic regression model. That is

$$GIC\,\;score=p=\frac{1}{1+e^{-\theta \left(p\right)}}$$

*E. Correlation analysis between GIC score and well-established measures of essential genes.* To explore the relationship between GIC score and several known measures of essential genes, we downloaded corresponding datasets described in detail below and got the intersections of GIC scores and each of them. To assess gene persistence, we counted the homolog number for each gene using data from the Homologene database (NCBI Resource Coordinators, 2016) (build 68). To evaluate sequence conservation, we retrieved the dN/dS ratio of each one-to-one mouse-human (and human-mouse) ortholog pair from the Ensembl database (release 83). The interaction network degrees were derived from the protein-protein interactions recorded in the BioGRID database (Stark et al., 2006) (release 3.4.135). At last genes were sorted by GIC score and median-binned into 200 bins for clearer illustration.

### Comparing the Accuracy of Human and Mouse Essential Gene Prediction

Gene essentiality was annotated as a Boolean value based on the corresponding essential gene set acquired from DEG. Using the R package pROC (Robin et al., 2011), the ROC curves were plotted and the AUC values for GIC score and the abovementioned measures were calculated and compared. Note that only the samples for which all of the above-mentioned measures were available were used during the comparison.

### Four Pairs of Genes for Further Validation of Candidate Gene Identification

Based on the transcriptomic data from PDGF-BB-treated rat aortic smooth muscle cells (Lee et al., 2010), we calculated FC value for each gene but did not perform statistical test to get *p*-value because there are only two samples for both the case and control. We then randomly selected four pairs of genes for further validation of candidate gene identification according to the following rules (**Supplementary File S1**). For each pair of genes, (1) one is with more significant expression change but less GIC score, the other is with less significant expression change but higher GIC score; (2) the expression of the two genes are at comparable level.

*H. Primary culture of rat vascular smooth muscle cells* – Aortic smooth muscle cells were isolated from male Sprague Dawley rats and cultured in DMEM medium supplemented with 20% FBS, 2 mM L-glutamine, 100 U/mL penicillin, and 10 mg/mL streptomycin. The media were renewed twice a week. All experimental procedures were conducted within a CO_2_ incubator at a temperature of 37°C, in an atmosphere of 95% air and 5% CO_2_.

### siRNA Knockdown of Target mRNAs in Primary Rat VSMCs

Primary rat VSMCs with the confluence of 60% were synchronized with serum-free starvation for 24 h, and then transfected with siRNA mixtures against various mRNAs (50 nM) or scrambled siRNA (50 nM) using VigoFect transfection kit (Vigorous Biotechnology, Cat No. T001) for 48 h. The siRNAs against each target mRNA were the mixture of four sets of sequences according to different part of target mRNA. All the siRNA sequences were designed and synthesized by Beijing Biolino Inc., All the siRNA sequences against various target mRNAs were provided in **Supplementary File S2**. The scrambled siRNA was also provided by Beijing Biolino Inc.

### Real Time PCR Analysis of Target mRNA Levels After siRNA Transfection

Forty eight hours post transfection, total cellular RNA was extracted using the Trizol reagent according to the manufacturer’s instructions. 0.5–1.0 μg of total RNA was used for the reverse transcription reaction. Quantitative real time PCR was performed using the DNA Engine with Chromo four Detector (MJ Research, Waltham, MA, United States). The relative expression of target genes in various groups were calculated using 2^-ΔΔCt^ methodology as detailed previously (Jia et al., 2014; Wang C. et al., 2014). β-actin mRNA had been used as housekeeping gene in the current study. All primer sequences used for real-time PCR assays were listed in **Supplementary File S3**.

*K. Cell viability assay* – Cell viability was measured by MTT assay. In brief, primary rat VSMCs were seeded and transfected in 24-well plates. At 48 h post siRNA transfection, MTT assays were performed. In each experiment, 3–4 observations were set and determined for each siRNA mixture. The average absorbance reflected cell viability with the data normalized to the control group.

### Cell Cycle Analysis

At 48 h post transfection, Primary rat VSMCs proliferation was evaluated by direct cell counting using a cytometer at indicated time point after treatment. Cells were harvested and stained with propidium iodide using a Cycle TEST PLUS DNA Reagent Kit (Becton Dickinson, United States). Cell cycles were analyzed using flow cytome- try with a FACScan (Becton Dickinson, United States).

### Code Availability

GIC is implemented in Python and it relies on the external program RNAfold. We provide convenient online service on our GIC web server^3^. However, as for large RNAs or batch jobs, we recommend users download the source code on this server. Besides, the pre-calculated GIC scores of human and mouse genes, including both mRNAs and lncRNAs, are also available on the server.

## Results

### The Construction of GIC

In brief, we managed to construct a logistic regression model (GIC) by integrating several features that can be derived from RNA sequences or predicted RNA secondary structures for measuring gene essentiality. First of all, the length of a RNA sequence was considered as a feature of gene essentiality based on the observation that RNAs encode conserved proteins are longer than those encode proteins with less conservation (Lipman et al., 2002). And then we integrated the frequencies of some specific nucleotide triplets into the model. In addition, we found mRNA products of essential genes often form more stable structures, which are found to influence gene expression (Wan et al., 2014). Thus, we utilized RNAfold (Hofacker et al., 1994) to predict RNA secondary structure and its MFE. Given that longer RNAs normally have lower MFE than shorter RNAs, we normalized MFE by sequence length in the model. Finally, given the serious imbalance between the numbers of essential genes and non-essential genes, we randomly selected a subset of negative samples (non-essential genes) to keep a balanced positive-to-negative ratio in the training dataset and trained the logistic regression model based on the balanced dataset. GIC score was defined as the probability output of the model.

### Comparison of GIC Method With Previous Computational Methods on Protein-Coding Genes

**FIGURE 1:**
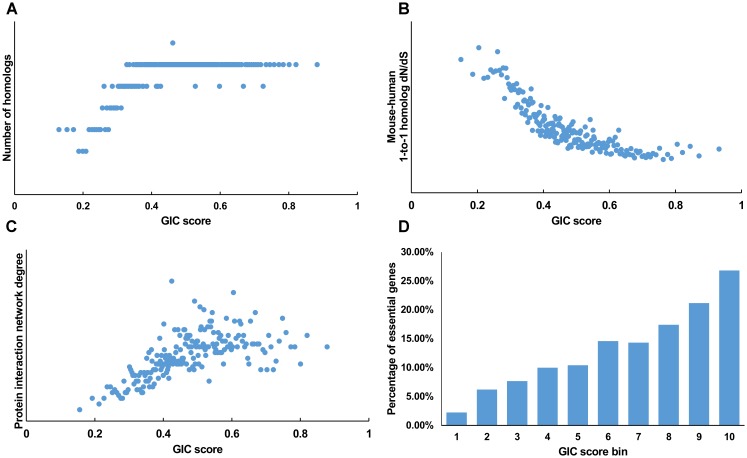
Correlation of GIC score with known measurements of essentiality in human genes. **(A)** Genes with higher GIC scores tend to have more homologs across species. **(B)** Genes with higher GIC scores tend to have slower evolutionary rate as measured by dN/dS ratio. **(C)** Proteins encoded by genes with higher GIC scores tend to have higher degrees in protein interaction network. **(D)** The percentage of human essential genes increases with GIC score.

**FIGURE 2:**
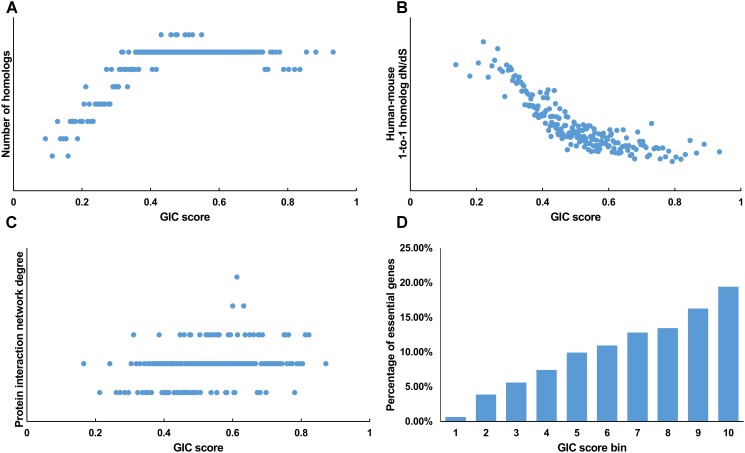
Correlation of GIC score with known measurements of essentiality in mouse genes. **(A)** Genes with higher GIC scores tend to have more homologs across species. **(B)** Genes with higher GIC scores tend to have slower evolutionary rate as measured by dN/dS ratio. **(C)** Proteins encoded by genes with higher GIC scores tend to have higher degrees in protein interaction network. **(D)** The percentage of human essential genes increases with GIC score.

We first tested GIC score on protein-coding genes. We observed harmonious correlations between human GIC scores and other computational scores (**Figures 1A–C**; Spearman *ρ* = 0.67, *P* = 6.17 × 10^-27^ with homolog number, Spearman *ρ* = ^-^0.92, *P* = 0 with dN/dS, Spearman *ρ* = 0.69, *P* = 4.51 × 10^-30^ with protein interaction network degree, respectively). For mouse genes, we got similar results (**Figures 2A–C**).

**FIGURE 3:**
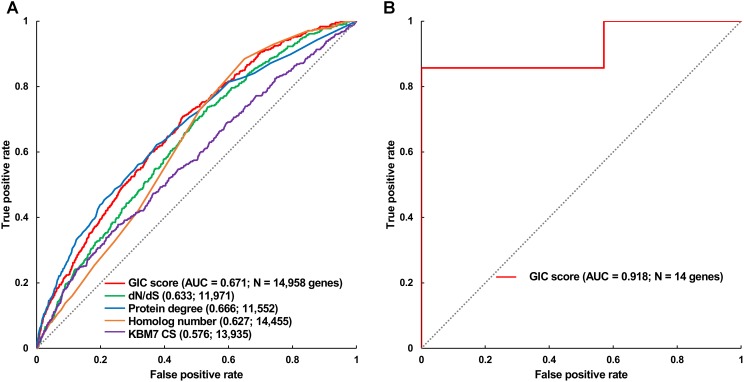
Validation of GIC score. **(A)** ROC curves illustrating the results from human essential gene prediction analysis. **(B)** ROC curves illustrating the results of essentiality prediction in an independent mouse lncRNA dataset.

Furthermore, we took the human and mouse essential genes stored in the DEG database as the benchmarks to evaluate the accuracy of GIC score. First, we ranked the human and mouse genes by GIC score and simply divided them into ten equal groups, respectively. Indeed, essential genes were enriched in groups of genes with higher GIC scores for both human (**Figure 1D**; *P* = 1.31 × 10^-69^, Pearson’s Chi-squared test) and mouse (**Figure 2D**; *P* = 7.80 × 10^-68^, Pearson’s Chi-squared test). Moreover, in terms of performance on the area under the receiver operator characteristic (ROC) curve (AUC), as for the testing set, GIC score (AUC = 0.671) outperformed both genetics method (**Figure 3A**) (CRISPR/Cas9 scores in KBM7 cell line (Wang et al., 2015), AUC = 0.576, *P* = 9.02 10-^7^, bootstrap test) and other computational methods, including homolog number (AUC = 0.628, *P* = 0.0026, bootstrap test), the dN/dS ratio of mouse-human 1-to-1 homolog (AUC = 0.633, *P* = 0.016, bootstrap test) and protein interaction network degree (AUC = 0.666, *P* = 0.78, bootstrap test). On the training set, human GIC score achieved an AUC of 0.675 with 10-fold cross validation, also better than the other scores (KBM7 CS, AUC = 0.569, *P* = 1.55 10^-20^, bootstrap test; homolog number, AUC = 0.629, *P* = 0.0001; dN/dS, AUC = 0.642, *P* = 0.0049; degree, AUC = 0.644, *P* = 0.026) (**Supplementary Figure S1**). For mouse, we got similar results (**Supplementary Figure S2**). Besides competitive prediction performance, moreover, GIC score only takes the information derived from RNA sequence, which makes it easier and more widely applicable than other computational methods.

### Performance of GIC Method on Predicting the Essentiality of lncRNAs

Next, we directly tested if GIC score is feasible to predict essential lncRNAs. To this end, we gleaned 14 mouse lncRNAs, of which seven were essential and the others were non-essential in mutagenesis assays, as an independent testing dataset (Methods). On this testing lncRNA dataset, GIC score showcased an AUC of 0.918 (**Figure 3B** and **Supplementary File S4**). Besides, we randomly selected seven mouse lncRNAs as negative replacements for 10,000 times and observed that the AUC values were larger than 0.85 and 0.75 in approximately 60 and 90% of the cases, respectively. The outcome again verified the viability of GIC score. Currently, there is no specific tool for essential lncRNA prediction, mainly due to the special characteristics of lncRNAs. Our GIC score can measure lncRNA essentiality with RNA sequence only and will serve as a promising tool to prioritize functionally important lncRNAs. It is interesting to check the GIC scores for some well established important lncRNAs. To do this, we focused on three famous lncRNAs (HOTAIR, H19, and MALAT1) which showed critical roles in a number of human diseases (Chen et al., 2013). As a result, all the three lncRNAs showed high GIC importance scores (HOTAIR: GIC = 0.483638027652, ranking = 97/1000; H19: GIC = 0.459905955417, ranking = 119/1000; MALAT1: GIC = 0.79923890709, ranking = 2/1000).

### Evaluating Hotspot Research Genes

**FIGURE 4:**
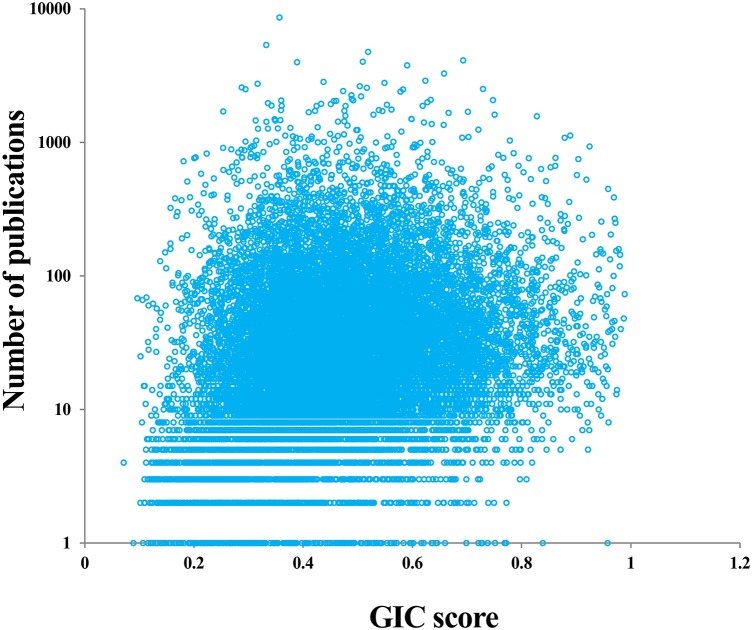
Correlation of GIC score and number of publications of genes.

It is interesting to investigate whether the genes with many publications (hotspot research genes) are really important or not. For doing so, we first counted the number of publications for each human gene based on the NCBI file of gene2pubmed. We then mapped each gene with GIC score and number of publications. As a result, we found a significant positive correlation between GIC score and number of publications (Rho = 0.23, *P*-value = 2.83e-221), suggesting that genes with more publications tend to be more essential. However, there are some genes with many publications have a small GIC score and some genes with less publications have a great GIC score (**Figure 4** and **Supplementary File S5**). For example, SCGB1A1 (secretoglobin family 1A member 1) has 150 publications but its GIC score is only 0.147, suggesting that it could be less important but attract many studies. On the other hand, NBPF20 (NBPF member 20) has a GIC score of 0.958 but has only one publication.

### Evaluating Cross-Species GIC Difference Between Human and Mouse

**FIGURE 5:**
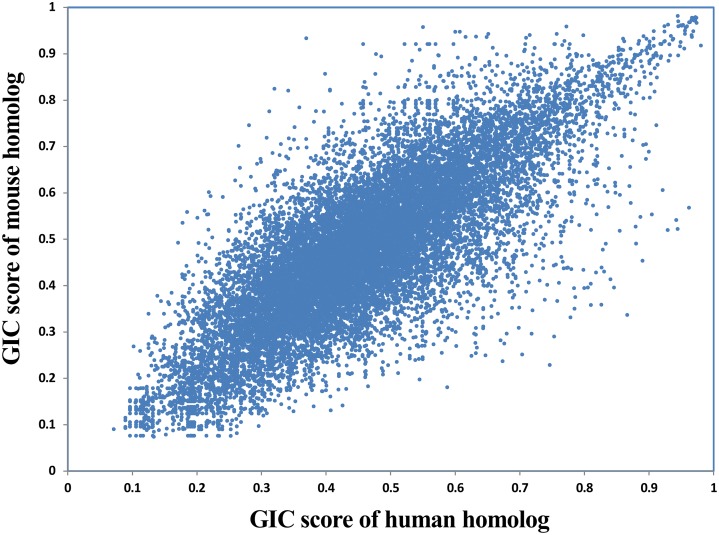
Correlation of GIC score of homologous genes between human and mouse.

Given that a lot of basic medical studies are performed on animal models, it is critical to dissect whether a gene in animal is representative for that in human body. GIC scores could provide clues to answer this question. Here we compared the GIC scores of homologous genes between human and mouse in a large scale. As a result, GIC score in human gene is significantly correlated with that mouse gene (Rho = 0.79, *P*-value = 0, Spearman’s correlation; **Figure 5**), suggesting that normally mouse genes are representative for human genes. However, there are indeed a number of genes which show big difference in importance score between human and mouse (**Supplementary File S6**). For example, DOK6 (docking protein 6) has a GIC score of 0.746 in human but 0.229 in mouse, whereas RAB3C (member RAS oncogene family) has a GIC score of 0.281 in human but 0.746 in mouse. These results suggest that it has a high risk of failure when performing medical studies on these genes from mouse models to human. It should be noted that sequence conservation score could be also used to dissect the difference of cross-species difference in homolog genes. However, GIC can provide more information, for example in which species the given homolog genes are more or less important.

### GIC Improves the Identification of Candidate Genes From Transcriptomic Data

**FIGURE 6:**
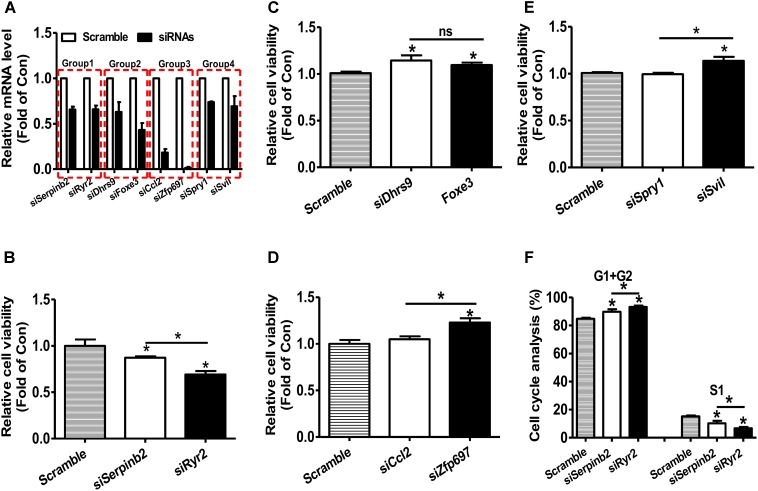
siRNA-mediated silencing of target mRNAs on the cell viability of primary rat VSMCs. **(A)** The efficacy of siRNA treatment on the repression of target mRNA levels. The target mRNA levels were analyzed by real time PCR assays at 48 h post siRNA transfection. *N* = 5, ^∗^*P* < 0.05 vs. control cells transfected with scrambled siRNAs. **(B–E)** Silencing of target mRNAs on the cell viability. At 48 h post siRNA transfection, cell viability was determined using MTT assay as described in experimental procedure. In every experiment, 3–4 parallel observations were set for each siRNA mixture. In panels **B–E**, GIC-predicted essential genes but with less significant expression change were presented as fill bars, whereas GIC-predicted non-essential genes but with significant expression change were presented as blank bars. *N* = 4, ^∗^*P* < 0.05 versus control cells transfected with scrambled siRNAs or between indicated two groups. **(F)**. Cell cycle analysis of primary rat VSMCs. Silencing of Serperb2 and Ryr2 on cell cycle determined by flow cytometry. *N* = 4, ^∗^*P* < 0.05, ^∗∗^*P* < 0.01 vs. control cells treated with scrambled siRNAs or between two indicated groups.

RNA-seq and microarray based transcriptomic profiling is becoming a basic technology in modern molecular biology and medicine (Cieslik and Chinnaiyan, 2018). One basic task is to identify the candidate genes, which is usually implemented by first computing fold change (FC) and/or *P*-value by statistical tests (e.g., *t*-test and wilcoxon test) and then comparing the FC value and *P*-value with their thresholds for each transcript. If one transcript passed the thresholds (for example FC > = 1.5 and/or *P*-value < 0.05), it will be identified as up-regulated gene if FC > 1 or down-regulated gene if FC < 1. The identification of the candidate gene signature that really represents the molecular phenotype of the interested biological process (e.g., disease, drug response etc) is a critically important task. However, the current strategy (FC and/or statistical *P*-value) does not consider the importance of the investigated transcripts. We hypothesizes that a important-but-not-such-significantly-differentially expressed gene (IBNS-DEG) may play important roles in the given biological process although it is not taken as a candidate gene; whereas a not-important-but–significantly-differentially expressed gene (NIBS-DEG) may be not important in the given biological process although it is taken as a candidate gene. Thus, the above popular strategy could produce a number of false positives (the wrongly identified candidate genes) and false negatives (the real candidate genes but not identified). Therefore, a quantitative GIC score could provide great helps in identifying candidate genes from a transcriptomic data. To test this hypothesis, four groups of genes with more significant expression change but lower GIC scores or with less significant expression change but higher GIC scores were selected and calculated based on the microarray data of rat vascular smooth muscle cell (VSMC) proliferation model (**Supplementary File S1**) (Lee et al., 2010). The effects of silencing these genes were then analyzed in primary rat VSMC. The efficacy of siRNA transfection on the target mRNA levels were shown in Figure, and silencing efficacy in each group was comparable (**Figure 6A**). In groups 1–3, Serpinb2, Dhrs9, and Cc12 were the genes with more significant upregulation but lower GIC scores, whereas Ryr2, Foxe3, and Zfp697 were those with less significant upregulation but higher GIC scores, respectively. In group 1, silencing of Ryr2 reduced more cell viability than silencing of Serpinb2 (**Figure 6B**). In group 2, silencing of both Dhrs9 and Foxe3 increased cell viability but there is no difference between them (**Figure 6C**). In group 3, silencing of Cc12 had little effect on cell viability, whereas silencing of Zfp697 significantly increased cell viability (**Figure 6D**). In group 4, Spry1 was the gene with more significant downregulation but lower GIC scores, whereas Svil was the one with less significant downregulation but higher GIC scores. In group 4, silencing of Spril failed to affect cell viability, whereas silencing of Svil significantly increased cell viability (**Figure 6E**). Cell cycle analyses in group 1 was further performed to validate the cell viability analyses data. Silencing of Ryr2 increased the cells in G1 and G2 phases, and reduced the cells in S1 phase than silencing of Serpinb2 (**Figure 6F**). These data further supported the findings that the cells with Ryr2 silencing exhibited less cell viability than those with Serpinb2 silencing (**Figure 6B**). Overall, these findings strongly supported the accuracy of GIC method in predicting the importance of genes. More importantly, GIC method provides a novel strategy for identifying candidate genes in transcriptomic data from RNA-seq and microarray, and extends largely the traditional strategy based only on expressional change.

## Discussion

Measuring gene essentiality is an important issue for both biology and medicine. Although traditional computational methods can evaluate gene essentiality, they are only feasible to a part of protein-coding genes. More importantly, they are not feasible to long noncoding RNAs (lncRNAs), a big class of genes in human genome. To overcome the above limitations, we defined GIC (Gene Importance Calculator) score on the basis of sequence information.

Overall, our data validated the competitive performance of GIC for quantifying essentiality of genes/lncRNAs. Moreover, GIC is feasible to all mRNAs and lncRNAs because it only needs sequence as input. In addition, we explored potential applications of GIC by several case studies. GIC can provide quantitative evaluation for the genes that are research hotspots and for the genes that are not investigated well. For basic medical studies from animal model to clinic, GIC can evaluate whether a gene in animal is representative for that in human, which could influence the success or failure of animal-human translation studies. Finally, GIC can provide helps in identifying candidate genes from transcriptomics. It should be noted that GIC computes MFE using the external program RNAfold, which has a limitation for RNA length (<20000 nt). Although only a small fraction of mRNAs and lncRNAs are longer than this length, GIC does not work on these RNAs. Recently, dissecting lncRNA-disease associations is becoming a important topic in bioinformatics (Chen and Yan, 2013; Chen et al., 2013, 2016, 2017; Chen, 2015), GIC cannot be used to predict the association for a given lncRNA with specific disease. But it can be used to evaluate the global association of an lncRNAs with human diseases. Normally, lncRNAs with greater importance score would be associated with more diseases. Given that the functions of many human protein-coding genes and lncRNAs are still awaiting exploration, our new method provides an effective strategy for identifying and characterizing new genes and lncRNAs with important functions, which definitely will shed light on the pathogenesis, diagnosis, and therapy of human diseases.

## Author Contributions

PZ and YZ implemented the algorithms and web-server. JC and YM performed the animal and cell experiments. PZ, JY, and QC drafted the manuscript. QC and JY conceived, designed, and supervised the study.

## Conflict of Interest Statement

The authors declare that the research was conducted in the absence of any commercial or financial relationships that could be construed as a potential conflict of interest.

## Footnotes

1 http://www.cuilab.cn/gic

2 http://www.informatics.jax.org/phenotypes.shtml

3 http://www.cuilab.cn/gic

## Supplementary Material

FILE S1 The characterics of selected genes. In each group, one gene with most significant expression change but lower GIC score and one gene with less significant expression change but higher GIC score were selected.

FILE S2 siRNA sequences against human target mRNAs.

FILE S3 List of oligonucleotide primer pairs used in real time RT-PCR analysis.

FILE S4 GIC scores of the independent testing set containing 14 mouse lncRNAs (#1–7: essential; #8–14: non-essential).

FILE S5 GIC score and number of publications of genes.

FILE S6 GIC score and its difference of homologous genes between human and mouse.

FIGURE S1 The performance of human GIC score using 10-fold cross validation.

FIGURE S2 Validation of mouse GIC score. **(A)** ROC curves illustrating the results from mouse gene essentiality prediction analysis. **(B)** The performance of mouse GIC score using 10-fold cross validation results.
