# Supplementary material for: Defining Essentiality Score of Protein-Coding Genes and Long Noncoding RNAs
Source: Front Genet. 2018 Oct 9;9:380. doi: 10.3389/fgene.2018.00380 (PMC6189311; doi:10.3389/fgene.2018.00380)
Supplement: FILE S1 — The characterics of selected genes. In each group, one gene with most significant expression change but lower GIC score and one gene with less significant expression change but higher GIC score were selected. [file Table_1.DOCX]

**Supplementary File 1. The characterics of selected genes.** In each group, one gene with most significant expression change but lower GIC score and one gene with less significant expression change but higher GIC score were selected.

| Groups | Gene | GIC score | Fold change in microarray |
| --- | --- | --- | --- |
| Group 1 | Serpinb2 | 0.29635 | 154.8259 |
|  | Ryr2 | 0.958593 | 1.418069 |
| Group 2 | Dhrs9 | 0.318174 | 51.92157 |
|  | Foxe3 | 0.910719 | 5.429209 |
| Group 3 | Ccl2 | 0.20043 | 17.15976 |
|  | Zfp697 | 0.833884 | 6.160886 |
| Group 4 | Spry1 | 0.28944 | 0.141976 |
|  | Svil | 0.775984 | 0.676186 |

***The expression change was reported in the study “**Lee MY, Garvey SM, Baras AS, Lemmon JA et al. Integrative genomics identifies DSCR1 (RCAN1) as a novel NFAT-dependent mediator of phenotypic modulation in vascular smooth muscle cells. Hum Mol Genet 2010 Feb 1;19(3):468-79.” , and it had been cited in the manuscript.
