# Supplementary material for: Defining Essentiality Score of Protein-Coding Genes and Long Noncoding RNAs
Source: Front Genet. 2018 Oct 9;9:380. doi: 10.3389/fgene.2018.00380 (PMC6189311; doi:10.3389/fgene.2018.00380)
Supplement: FILE S2 — siRNA sequences against human target mRNAs. [file Table_2.DOCX]

**Supplementary File 2. siRNA sequences against human target mRNAs**

| Gene name | Species | siRNA number | SenseSeq |
| --- | --- | --- | --- |
| Serpinb2 | Rat | siRNA-1 | 5'-GCAGUAGACUUCCUUGAAUTT-3' |
|  | Rat | siRNA-2 | 5'GCUGAACAUUGGAUACAUATT-3' |
|  | Rat | siRNA-3 | 5'-CCUAGAACUUCCGUAUAUUTT-3' |
|  | Rat | siRNA-4 | 5'-CCUGAUGAGAUCGAAGAUUTT-3' |
| Ryr2 | Rat | siRNA-1 | 5'-CCAUACAUCCUGCCUCUAATT-3' |
|  | Rat | siRNA-2 | 5'-GCAGACAUCUACAUACUAUTT-3' |
|  | Rat | siRNA-3 | 5'-GCAAGGAGCUAAGCACUUATT-3' |
|  | Rat | siRNA-4 | 5'-GCUGAUGAUCCGAGGAUUATT-3' |
| Dhrs9 | Rat | siRNA-1 | 5'-GCUCGAGGGCGUGUUAUUATT-3' |
|  | Rat | siRNA-2 | 5'-GGUUUCAAUGACAGCUUAATT-3' |
|  | Rat | siRNA-3 | 5'-GCAGAUCCAAUAAAGACAATT-3' |
|  | Rat | siRNA-4 | 5'-CCAACCCAGAGAAUGUCAATT-3' |
| Foxe3 | Rat | siRNA-1 | 5'-GCUUCAUCACCGAGCGCUUTT-3' |
|  | Rat | siRNA-2 | 5'-CCUUUCCCUACGCGCCCUUTT-3' |
|  | Rat | siRNA-3 | 5'-GCAGCCAUCUACCGCUUCATT-3' |
|  | Rat | siRNA-4 | 5'-GCAAGCGCUUUAAGCGCACTT-3' |
| Ccl2 | Rat | siRNA-1 | 5'-GGGCCUGUUGUUCACAGUUTT-3' |
|  | Rat | siRNA-2 | 5'-GCAUCAACCCUAAGGACUUTT-3' |
|  | Rat | siRNA-3 | 5'-GGACUUCAGCACCUUUGAATT-3' |
|  | Rat | siRNA-4 | 5'-CCAUAAAUCUGAAGCUAAUTT-3' |
| Zfp697 | Rat | siRNA-1 | 5'-CCACAGGAUGAUGACCUAATT-3' |
|  | Rat | siRNA-2 | 5'-CCGCUACAAGACGCACCUUTT-3' |
|  | Rat | siRNA-3 | 5'-GGAUUCCAACCCACAGGAUTT-3' |
|  | Rat | siRNA-4 | 5'-GCCUGGAGCCCUUCGGUUUTT-3' |
| Spry1 | Rat | siRNA-1 | 5'-CCGGCAGAGGUUAGACUAUTT-3' |
|  | Rat | siRNA-2 | 5'-GGACCUUCGGUAGCGAGAATT-3' |
|  | Rat | siRNA-3 | 5'-GCGGUGUUGGUCUUCACAUTT-3' |
|  | Rat | siRNA-4 | 5'-GGACACGGUAGUUGACAUUTT-3' |
| Svil | Rat | siRNA-1 | 5'-GCUCCCAAGUUGACAUCUUTT-3' |
|  | Rat | siRNA-2 | 5'-GCAUCUGAACUUGCGACAUTT-3' |
|  | Rat | siRNA-3 | 5'-GGAAGUCACGUUAGCACAATT-3' |
|  | Rat | siRNA-4 | 5'-GGAGCCACCCUGCUUCCUUTT-3' |
