# Supplementary material for: Defining Essentiality Score of Protein-Coding Genes and Long Noncoding RNAs
Source: Front Genet. 2018 Oct 9;9:380. doi: 10.3389/fgene.2018.00380 (PMC6189311; doi:10.3389/fgene.2018.00380)
Supplement: FILE S3 — List of oligonucleotide primer pairs used in real time RT-PCR analysis. [file Table_3.DOCX]

**Supplementary File 3. List of oligonucleotide primer pairs used in real time RT-PCR analysis.**

| Gene name | species | Forward | Reverse |
| --- | --- | --- | --- |
| β-actin | Human | 5'-ACTCTTCCAGCCTTCCTTCC-3' | 5'-TCTCCTTCTGCATCCTGTCG-3' |
| Serpinb2 | Rat | 5'-CAGTAGATGTGAACGAGGAGG-3' | 5'-GTATGGTGCGGGTAATGT-3' |
| Ryr2 | Rat | 5'-CTGCCCACTGTTGACTTG-3' | 5'-CTGTTCTGCTTGTCCTCG-3' |
| Dhrs9 | Rat | 5'-CAGGCTTCGGAAACTTAG-3' | 5'-GCAGTCTCCTTGACATTCTC-3' |
| Foxe3 | Rat | 5'-CGCTCTTATCGCTATGGC-3' | 5'-TCCAGTAGTTGCCCTTGC-3' |
| Ccl2 | Rat | 5'-GCAGGTGTCCCAAAGAAG-3' | 5'-GTGCTTGAGGTGGTTGTG-3' |
| Zfp697 | Rat | 5'-CCGCAACACTTACCTGAC-3' | 5'-AGCAAGTGCGAACTGAGG-3' |
| Spry1 | Rat | 5'-ATGGATTCCCCAAGTCAG-3' | 5'-GTCCCTGTCATAGTCTAACCTC-3' |
| Svil | Rat | 5'-CCCACCGAAAACAAGATAAC-3' | 5'-CCAGCATGGATAAGGTAAGAC-3' |
