# Supplementary material for: Defining Essentiality Score of Protein-Coding Genes and Long Noncoding RNAs
Source: Front Genet. 2018 Oct 9;9:380. doi: 10.3389/fgene.2018.00380 (PMC6189311; doi:10.3389/fgene.2018.00380)
Supplement: FILE S4 — GIC scores of the independent testing set containing 14 mouse lncRNAs (#1–7: essential; #8–14: non-essential). [file Table_4.DOCX]

**Supplementary File 4.** GIC scores of the independent testing set containing 14 mouse lncRNAs (#1-7: essential; #8-14: non-essential).

| **#-Name** | **GIC score** | **Importance ranking (‰)** |
| --- | --- | --- |
| 1-Tsix | 0.631696136 | 25 |
| 2-Dnm3os | 0.551314847 | 55 |
| 3-Gt(ROSA)26Sor | 0.317037271 | 482 |
| 4-Fendrr | 0.43895292 | 154 |
| 5-Mir124a-1hg | 0.536117294 | 62 |
| 6-Haglr | 0.492569368 | 92 |
| 7-Meg3 | 0.553522394 | 54 |
| 8-Ptgs2os2 | 0.303786189 | 535 |
| 9-Mannr | 0.185201825 | 905 |
| 10-Hottip | 0.398189216 | 229 |
| 11-Lncenc1 | 0.154694352 | 951 |
| 12-Trp53cor1 | 0.390004747 | 248 |
| 13-Kantr | 0.394459863 | 238 |
| 14-Celrr | 0.351835765 | 359 |
