# Supplementary figures and images for: Defining Essentiality Score of Protein-Coding Genes and Long Noncoding RNAs
Source: Front Genet. 2018 Oct 9;9:380. doi: 10.3389/fgene.2018.00380 (PMC6189311; doi:10.3389/fgene.2018.00380)

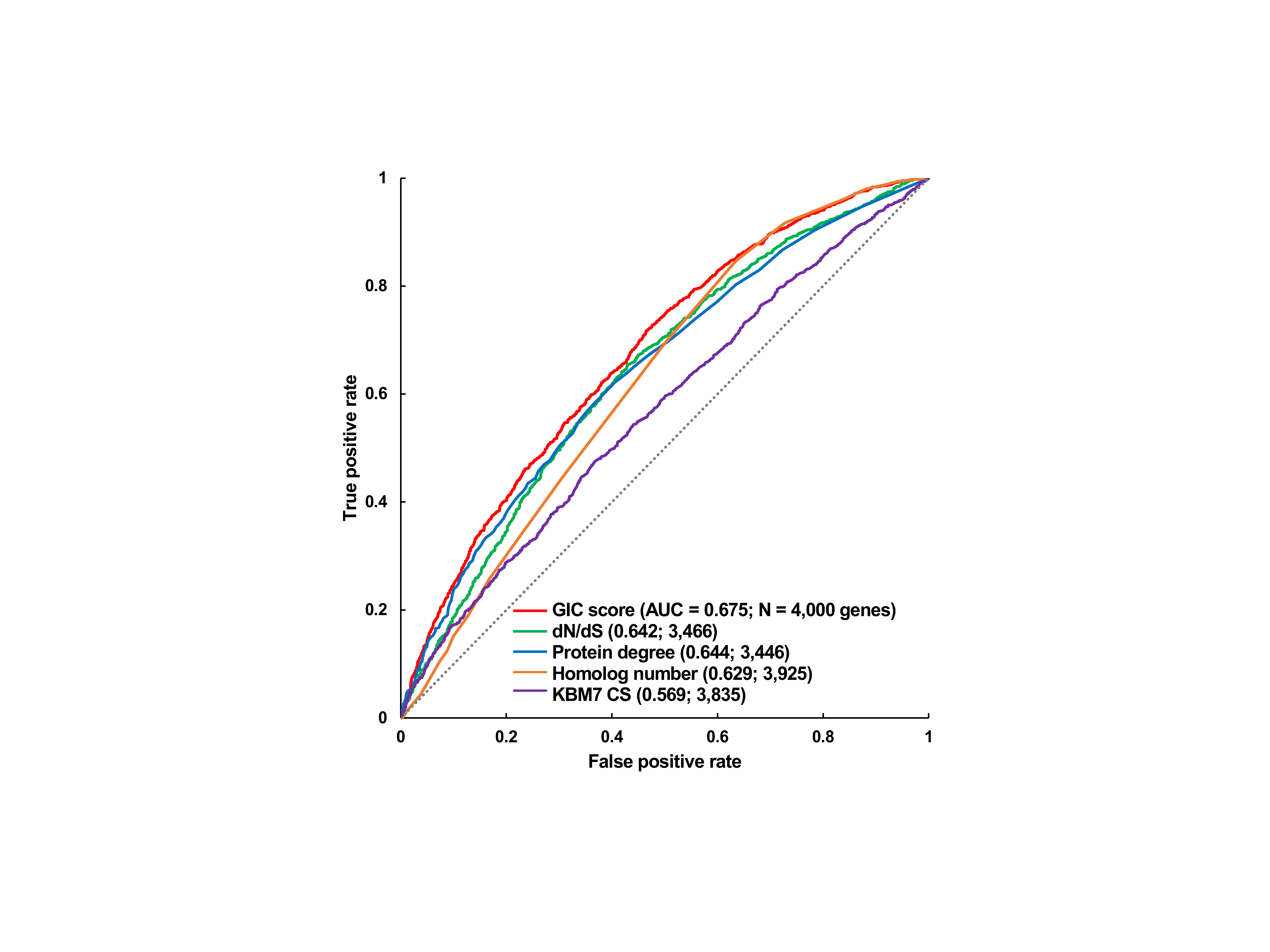

Supplement: FIGURE S1 — The performance of human GIC score using 10-fold cross validation. [file Image_1.TIF]

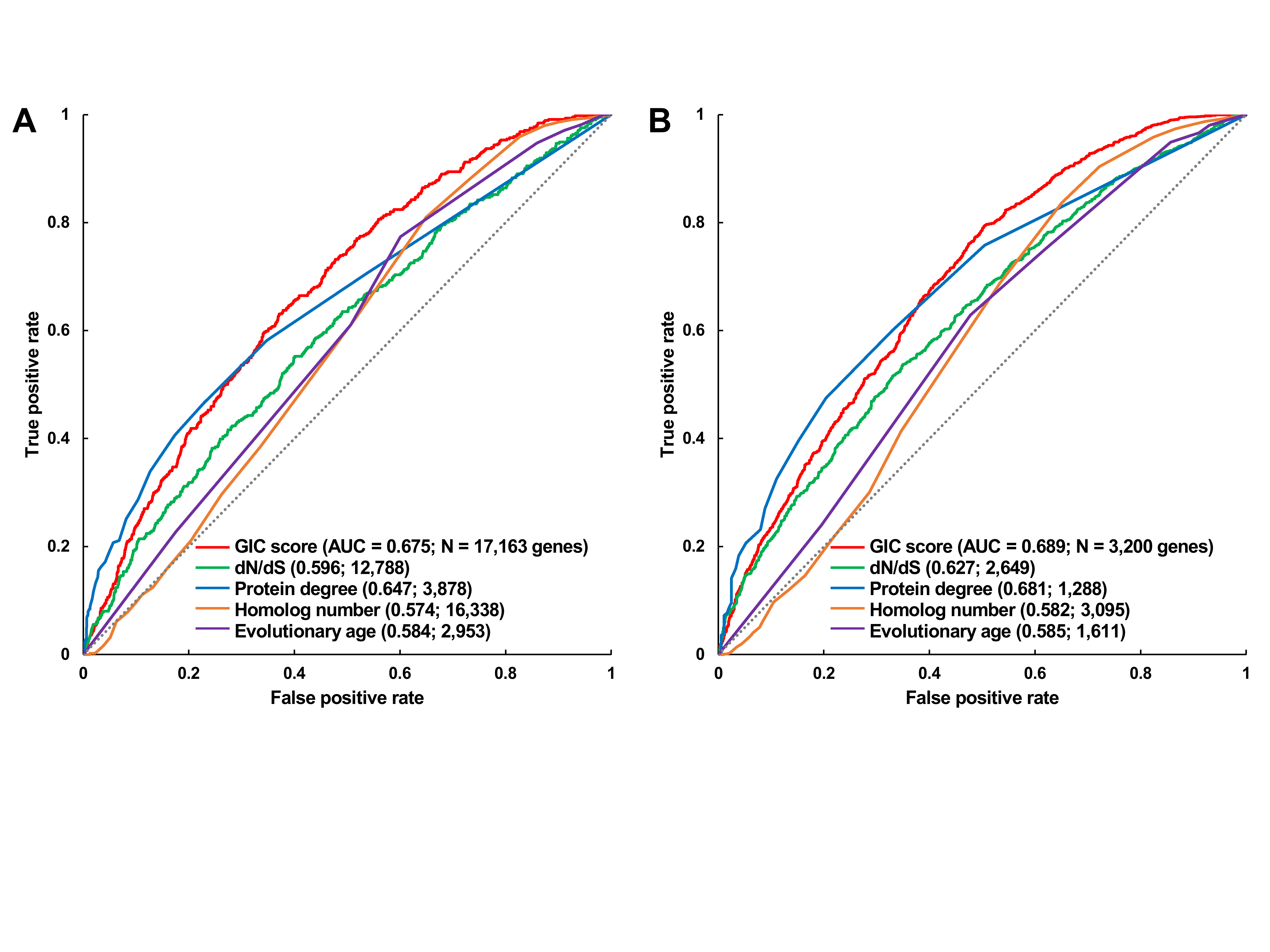

Supplement: FIGURE S2 — Validation of mouse GIC score. (A) ROC curves illustrating the results from mouse gene essentiality prediction analysis. (B) The performance of mouse GIC score using 10-fold cross validation results. [file Image_2.TIF]
